# Quantitative iodine-mapping of photon-counting CT detects large-vessel vasculitis: A case report

**DOI:** 10.1016/j.radcr.2026.05.037

**Published:** 2026-06-03

**Authors:** Steffen Lückerath, André Euler

**Affiliations:** Department of Radiology, Kantonsspital Baden, affiliated Hospital for Research and Teaching of the Faculty of Medicine of the University of Zurich, Baden, Switzerland

**Keywords:** Photon-counting CT, Spectral CT, Aorta, Vasculitis, Iodine, Giant cell arteritis, Case report

## Abstract

Diagnosis of large-vessel vasculitis can be challenging because clinical symptoms and early imaging findings are often nonspecific. A 69-year-old woman presented to the emergency department with severe headache and markedly elevated inflammatory markers. Initial brain CT, brain MRI, and ultrasound examinations revealed no significant findings. On repeat presentation, photon-counting-detector CT (PCCT) was performed to search for an infectious focus and demonstrated concentric mural thickening of the aorta and supra-aortic branches. Retrospectively reconstructed iodine maps, exploiting the intrinsic spectral capabilities of PCCT, showed increased iodine concentration within the aortic wall, raising suspicion of active large-vessel vasculitis. The diagnosis of giant cell arteritis was subsequently corroborated by MRI, ^18^FDG PET/CT, and repeat ultrasound of the temporal artery. Corticosteroid therapy led to rapid clinical and biochemical improvement. PCCT with spectral postprocessing may provide valuable additional information for the early detection of large-vessel vasculitis, particularly in cases with initially inconclusive imaging.

## Introduction

Large-vessel vasculitis may present with nonspecific symptoms and elevated inflammatory markers, while initial imaging findings can be subtle or absent [1,2]. Early diagnosis is key to prevent complications and initiate immunosuppressive therapy. Cross-sectional imaging modalities such as MRI and ^18^F-FDG PET/CT play an established role in confirming vascular inflammation but are not always readily available or immediately performed.

Photon-counting detector CT (PCCT) [3] is an emerging technology that enables inherent spectral imaging without the need for dedicated prospective dual-energy CT protocol selection. By allowing retrospective material decomposition, PCCT provides quantitative iodine maps [4]. This may aid in differentiating inflammatory vascular wall enhancement from noninflammatory atherosclerotic changes but may also provide further information about the severity of inflammation. This case illustrates the value of PCCT iodine mapping as an on-demand problem-solving tool in large-vessel vasculitis.

## Case presentation

A 69-year-old woman presented to the emergency department with a 10-day history of intermittent severe headache (Visual Analogue Scale 7/10) and markedly elevated inflammatory markers (C-reactive protein 189 mg/L). The patient had no co-morbidities and no medical treatment in her medical history. She reported neither visual disturbances nor jaw claudication. There were no focal neurological deficits and no signs of infection on physical examination. Initial noncontrast CT of the head and CT angiography of the head and neck revealed no significant findings. Subsequent brain MRI and ultrasound of the temporal arteries did not show signs of vasculitis. Analgesic therapy led to symptomatic improvement and consequently the patient was discharged.

Four days later, the patient returned with persistent systemic inflammation and ongoing clinical symptoms. To search for a potential infectious focus, PCCT (NAEOTOM Alpha, Siemens Healthineers, Forchheim, Germany) of the chest and abdomen in venous phase was performed. The scan protocol included intravenous administration of 80 mL of iodinated contrast material (Iopamidol 370 mg I/mL) followed by a saline chaser of 30 mL, both at an injection rate of 4.0 mL/s.

Thoracic imaging was acquired with a pitch of 3.2 and a gantry rotation time of 0.25 s at 120 kV and 35 effective mAs, resulting in a volume CT dose index (CTDI_vol_) of 2.8 mGy. Abdominal imaging was performed using a pitch of 0.8 at 140 kV and 30 effective mAs, with a CTDI_vol_ of 3.44 mGy. The thoracic images were reconstructed as virtual monoenergetic images at 65 keV and were obtained in ultra-high-resolution (UHR) mode. The abdominal images were reconstructed as virtual monoenergetic images at 70 keV.

**Fig. 1:**
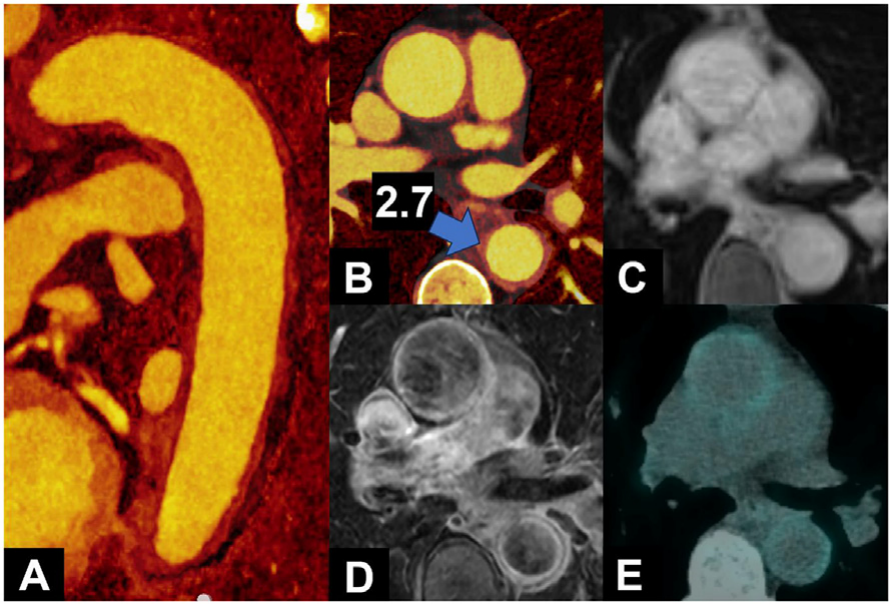
PCCT images of the thoracic aorta in a 69-year-old woman in venous phase. (A) and (B) color-coded iodine maps demonstrate circumferential wall thickening of the thoracic aorta with a quantitative iodine wall uptake of 2.7 mg I/mL. (C) T1-weighted late enhancement and (D) Dark blood images confirmed the circumferential wall thickening and increased gadolinium uptake of the thoracic aorta. (E) Subsequent ^18^ F-FDG-PET/CT confirmed increased tracer uptake indicative of large vessel vasculitis.

The examination incidentally demonstrated concentric wall thickening of the thoracic and abdominal aorta as well as the supra-aortic branches. Exploiting the intrinsic spectral capabilities of PCCT, iodine maps were retrospectively reconstructed (syngo.via, Siemens Healthineers, Forchheim, Germany). Iodine uptake of the aortic wall was measured by placing regions of interest (ROIs) within the thickened aortic wall while avoiding the vessel lumen. Measurements were performed along short vessel segments and averaged across multiple ROIs. Quantitative analysis revealed an increased iodine uptake within the aortic wall of up to 2.7 mg I/mL, favoring active vascular inflammation over noninflammatory atherosclerotic wall thickening (Fig. 1).

Subsequent contrast-enhanced MRI confirmed circumferential mural thickening and enhancement of the aorta and its major branches. ^18^F-FDG PET/CT demonstrated corresponding increased tracer uptake, consistent with active aortitis. Repeat ultrasound of the temporal arteries revealed wall thickening of the parietal and frontal branches, supporting the diagnosis of giant cell arteritis.

**Fig. 2:**
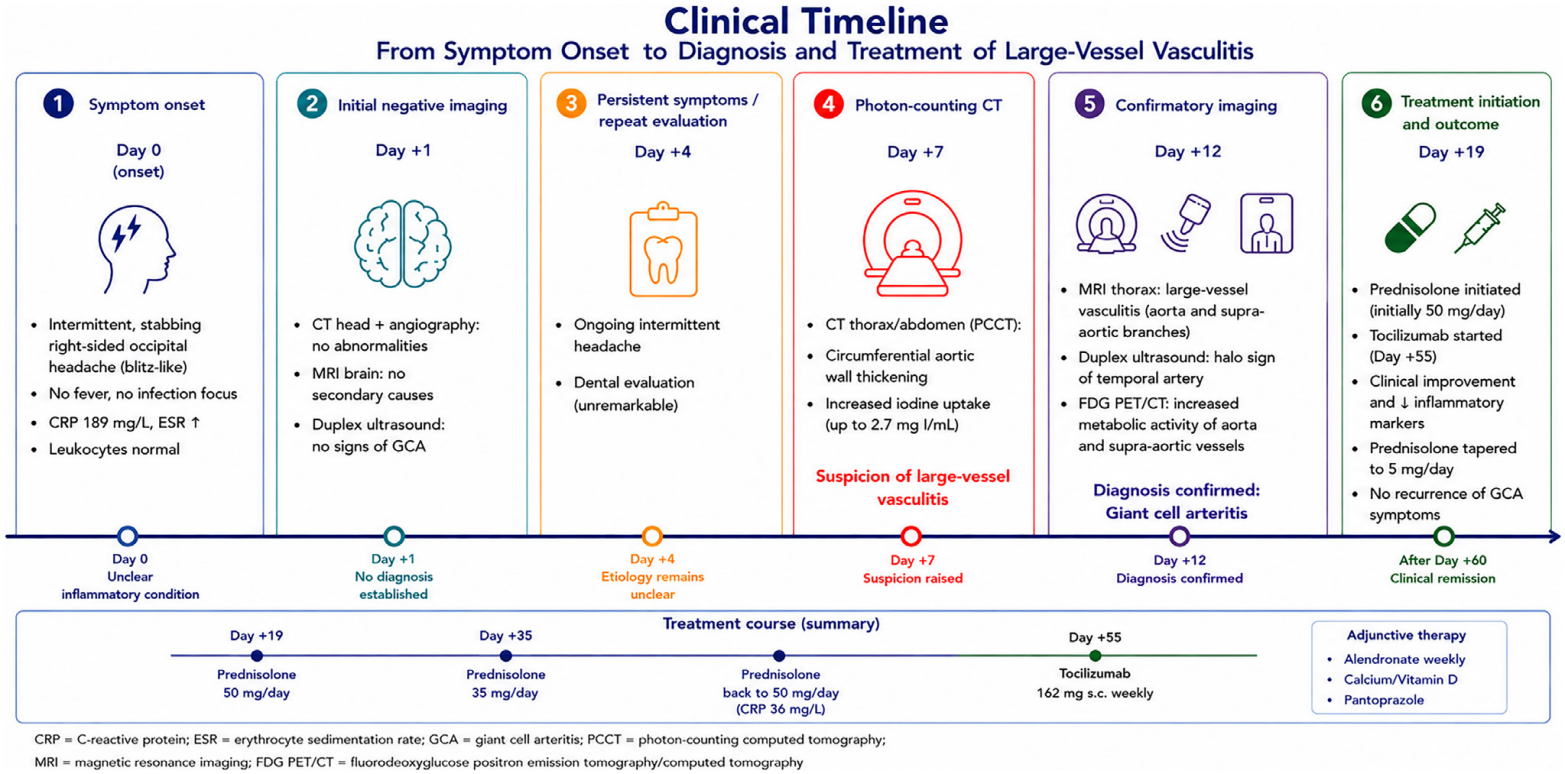
Clinical timeline of diagnostic work-up and treatment. Initial imaging was unremarkable despite elevated inflammatory markers. Photon-counting CT revealed increased iodine uptake in the aortic wall, leading to the diagnosis of large-vessel vasculitis and subsequent treatment initiation.

Corticosteroid therapy was initiated immediately after CT-based suspicion of large-vessel vasculitis, 4 days after the initial presentation, starting with Prednisolone 50 mg/d. Despite initial treatment, inflammatory markers remained elevated, with CRP decreasing from 189 mg/L at presentation to 36 mg/L, prompting temporary dose escalation followed by gradual tapering. Approximately 6 weeks later, additional immunosuppressive therapy with Tocilizumab (162 mg/wk) was initiated, allowing further reduction of corticosteroid dosage (Fig. 2).

Over a follow-up period of 2 months, the patient showed complete clinical remission with normalization of inflammatory markers. At the most recent follow-up, corticosteroid dosage had been successfully reduced to 5 mg/d under Tocilizumab therapy, with no recurrence of symptoms. Follow-up imaging has not yet been performed but is planned as part of routine disease monitoring.

## Discussion

This case demonstrates the diagnostic advantages of quantitative iodine-mapping using photon-counting detector CT for the detection of large-vessel vasculitis. While conventional CT primarily provides morphological information, PCCT enables retrospective material decomposition without the need for prospective dual-energy CT protocol selection.

Differentiating inflammatory wall enhancement from atherosclerotic changes can be challenging, particularly when imaging findings are subtle or incidentally detected. Quantitative iodine measurements may increase diagnostic confidence. Importantly, this information can be obtained without prior planning of a spectral imaging protocol, highlighting the flexibility of PCCT in routine clinical practice and emergency setting.

Differential diagnoses of arterial wall thickening include atherosclerosis and infectious aortitis. Atherosclerotic changes typically present as focal, eccentric wall thickening often associated with calcifications, rather than the smooth, circumferential involvement observed in this case. Infectious aortitis represents an important differential diagnosis, as it may also demonstrate increased contrast enhancement and iodine uptake due to inflammatory hyperemia as well as increased metabolic activity on ^18^F-FDG PET/CT. However, infectious aortitis usually presents with systemic signs of infection such as high fever and thoracoabdominal pain, often accompanied by focal or asymmetric wall involvement, perivascular inflammatory changes like soft tissue stranding, or abscess formation on imaging. None of these features were present in this patient, who remained afebrile and had no perivascular inflammatory changes on imaging.

In the present case, PCCT showed an iodine uptake of up to 2.7 mg I/mL. Normal aortic walls are expected to show only minimal iodine uptake. However increased iodine concentration has been reported in pathological vessel wall conditions, particularly in atherosclerotic coronary plaques, where enhancement is, however, typically focal and heterogeneous. Notably, there is evidence in the literature that lipid-rich or “soft” plaques may also demonstrate contrast enhancement [5]. However, standardized reference values or cut-off thresholds for iodine uptake in either atherosclerotic disease or large-vessel vasculitis are currently lacking and the quantitative findings should be considered exploratory. In our institutional experience, exploratory measurements in atherosclerotic plaques have shown iodine uptake values of up to approximately 1-1.5 mg I/mL, suggesting that the higher values observed in this case support the inflammatory origin. Using retrospective iodine-mapping, the diagnosis could be suggested quickly, expediting escalation to dedicated vascular imaging and enabling early initiation of corticosteroid therapy. PCCT iodine mapping could serve as a complementary problem-solving tool in addition to the established imaging modalities such as MRI or FDG PET/CT in inflammatory vascular imaging. As PCCT becomes more widely available, its role in vascular imaging may expand beyond high spatial resolution to include functional tissue characterization.

## Conclusion

Photon-counting detector CT with intrinsic iodine-mapping can facilitate the incidental detection and characterization of large-vessel vasculitis. Quantitative assessment of vascular wall iodine uptake may serve as a valuable problem-solving tool, supporting timely diagnosis and management even when vasculitis is not initially suspected.

## Patient consent

Informed consent was obtained for patient information to be published in this article.
